# Analysis of personal and national factors that influence depression in individuals during the COVID-19 pandemic: a web-based cross-sectional survey

**DOI:** 10.1186/s12992-020-00650-8

**Published:** 2021-01-05

**Authors:** Ji Ho Lee, Hocheol Lee, Ji Eon Kim, Seok Jun Moon, Eun Woo Nam

**Affiliations:** 1 Department of Health Administration, Yonsei University Graduate School, Wonju, Republic of Korea; 2 Yonsei Global Health Center, Yonsei University, Wonju, Republic of Korea; 3 Korea Institute for Health and Social Affair, Sejong City, Republic of Korea; 4 Health Administration Department, Yonsei University, Wonju, Republic of Korea

**Keywords:** COVID-19, Depression, Government response stringency index, Mental health

## Abstract

**Background:**

The World Health Organization (WHO) declared coronavirus disease (COVID-19) a pandemic on March 11, 2020. Previous studies of infectious diseases showed that infectious diseases not only cause physical damage to infected individuals but also damage to the mental health of the public. Therefore this study aims to analyze the factors that affected depression in the public during the COVID-19 pandemic to provide evidence for COVID-19-related mental health policies and to emphasize the need to prepare for mental health issues related to potential infectious disease outbreaks in the future.

**Results:**

This study performed the following statistical analyses to analyze the factors that influence depression in the public during the COVID-19 pandemic. First, to confirm the level of depression in the public in each country, the participants’ depression was plotted on a Boxplot graph for analysis. Second, to confirm personal and national factors that influence depression in individuals, a multi-level analysis was conducted. As a result, the median Patient Health Questionnaire-9 (PHQ-9) score for all participants was 6. The median was higher than the overall median for the Philippines, Indonesia, and Paraguay, suggesting a higher level of depression. In personal variables, depression was higher in females than in males, and higher in participants who had experienced discrimination due to COVID-19 than those who had not. In contrast, depression was lower in older participants, those with good subjective health, and those who practiced personal hygiene for prevention. In national variables, depression was higher when the Government Response Stringency Index score was higher, when life expectancy was higher, and when social capital was higher. In contrast, depression was lower when literacy rates were higher.

**Conclusions:**

Our study reveals that depression was higher in participants living in countries with higher stringency index scores than in participants living in other countries. Maintaining a high level of vigilance for safety cannot be criticized. However, in the current situation, where coexisting with COVID-19 has become inevitable, inflexible and stringent policies not only increase depression in the public, but may also decrease resilience to COVID-19 and compromise preparations for coexistence with COVID-19. Accordingly, when establishing policies such as social distancing and quarantine, each country should consider the context of their own country.

## Introduction

Coronavirus disease (COVID-19), first found in Wuhan, Hubei province, China on December 31, 2019, has led to 4,618,821 confirmed cases and 311,847 deaths worldwide in approximately 4 months, as of May 18, 2020 [1]. Thus, the World Health Organization (WHO) declared COVID-19 a pandemic, which is the highest level of alert for infectious diseases, on March 11. During such a pandemic, countries have implemented various policies to prevent the spread of COVID-19, such as the closure of schools, workplaces, and public transit, social distancing, and restriction of international and domestic travel.

The argument that infectious diseases influence the mental health of the public existed before the COVID-19 pandemic. Pfefferbaum reported that H1N1 caused a public health crisis [2], which influenced the public and local communities. In one study, Douglas found that mental health should be included in preparation strategies for seasonal influenza and that infectious diseases may cause serious mental health issues [3].

As with previous infectious diseases, studies on COVID-19 reported that it not only causes physical damage to the infected individuals but also damage to the mental health of the public. One study reported that vicarious trauma is more severe in nurses who do not directly contact COVID-19 patients and in the public rather than in nurses who directly contact COVID-19 patients [4]. Moreover, Wang who investigated psychological responses of the public to COVID-19 showed that one-third of their survey respondents reported experiencing anxiety [5].

The psychological influences of the COVID-19 pandemic on the public are not limited to China, where the disease originated, and it has become a worldwide problem. A report by the United Nations on May 13 suggested that mental health responses are necessary during the COVID-19 pandemic and recommend three approaches as follows: social integration approach for promotion, protection, and management of mental health; improved access to emergency mental health support measures; and establishment of a service system for recovery of mental health issues caused by COVID-19. The WHO selected 11 mental health rules and published them on their website, and UK National Health Service (NHS), US Centers for Disease Control and Prevention, and public health departments of the EU, Canada, and France have also published coping methods to manage COVID-19 stress in the public [1].

Therefore, this study aims to analyze the factors that affect depression in the public during the COVID-19 pandemic to provide evidence for COVID-19-related mental health policies and emphasize the need to prepare for mental health issues related to potential infectious disease outbreaks. The following are specific purposes: First, to confirm the level of depression in the public in each country during the COVID-19 pandemic. Second, to confirm personal factors that influence depression in individuals. Third, to confirm national factors that influence depression in individuals. To Conduct this study, we selected nine countries as our research area, where we have our overseas cooperator.

### Current state of COVID-19 infections in each country

Table 1 summarizes the population, number of confirmed COVID-19 cases, number of deaths, and numbers of confirmed COVID-19 cases and deaths per 100,000 in each country. The number of confirmed cases was highest in Peru, followed by China, Indonesia, Japan, and the Philippines. However, the number of confirmed cases per 100,000 was highest in Peru, followed by South Korea, the Philippines, Japan, and Paraguay. The number of deaths was highest in China, followed by Peru, Indonesia, the Philippines, and Japan, and the number of deaths per 100,000 was highest in Peru, followed by the Philippines, Japan, Indonesia, and South Korea.

**Table 1:** Comparison of population, confirmed cases, and deaths in each country (data retrieved on May 29, 2010) Source: WHO

Country	Population (number)	Confirmed cases (number)	Confirmed cases per 100,000 (number)	Deaths (number)	Deaths per 100,000 (number)
**South Korea**	51,269,000	11,344	22.126	269	0.525
**China**	1,439,324,000	84,547	5.874	4645	0.323
**Japan**	126,476,000	16,683	13.191	867	0.686
**Philippines**	109,581,000	15,049	13.733	904	0.825
**Indonesia**	273,524,000	24,538	8.971	1496	0.547
**Peru**	32,972,000	129,751	393.519	3788	11.489
**Paraguay**	7,113,000	884	12.428	11	0.155
**Ethiopia**	114,964,000	731	0.636	6	0.005
**Democratic Republic of Congo**	89,561,000	2659	2.969	68	0.076

### National indices for each country

Table 2 summarizes the distribution of national indices for countries in which the participants lived.

**Table 2:** National indices for each country

National indices	Stringency index score	Life expectancy	Literacy rate	Social capital	PPP 1,000,000 USD)
Country					
**South Korea**	77	82.63	97.96	42.482	2,029,000
**China**	57	76.47	96.35	57.248	23,160,000
**Japan**	56	84.10	99	44.448	5,429,000
**Philippines**	93	70.95	96.61	59.318	875,600
**Indonesia**	71	71.28	95.43	73.431	3,243,000
**Peru**	92	76.29	94.37	42.345	424,400
**Paraguay**	92	73.99	95.53	53.697	68,330
**Ethiopia**	75	65.87	49.03	46.814	200,200
**Democratic Republic of Congo**	81	60.03	77.22	38.740	28,880

## Methods

### Study design

This is a cross-sectional study that assessed the influence of national indices in each country concerning depression in individuals during the COVID-19 pandemic. To confirm depression in individuals and personal indices, a survey was conducted between May 25 and June 24, 2020. The survey was conducted in nine countries (South Korea, China, Japan, Philippines, Indonesia, Peru, Paraguay, Democratic Republic of Congo, and Ethiopia), and the participants were citizens of these nine countries. The survey questions were selected through expert meetings comprising public health experts at the Yonsei Global Health Center (YGHC), and content validity was confirmed through pre-tests in 10 individuals. We had original English questionnaire, and translated into 5 different languages: Korean, Chinese, Japanese, French and Spanish by foreign researcher in our center. The questions were reviewed by local professors and experts in each country to produce questionnaires that are suitable for each country. The minimum sample size for an effect size of 0.15, power of testing of 0.95, and a significance level of 0.05 calculated through G*Power 3.1 was 963. No participant withdrew during the survey or had missing answers. Thus, a total of 2683 individuals in the nine countries were surveyed. The online survey was distributed through Google Survey and Naver Online Survey Form. Snowball sampling was used for sample extraction. For this, cooperators were selected at institutions in each country, and the survey questionnaire was distributed.

### Instruments

#### Dependent variables

We use level of depression as dependent variable of this study. We used the Patient Health Questionnaire-9 (PHQ-9) to investigate level of depression in the participants. PHQ-9 was developed for diagnosing major depressive disorder and consists of nine questions. Each questionnaire checked the frequency of bothered by questionnaire and scored from 0(not at all) to 3(Nearly every day). As a result, PHQ-9 score ranging 0 to 27 by adding up the response of nine questions. PHQ-9 diagnosis depression in five categories: 0–4: no depressive symptoms, 5–9: mild, 10–14 moderate, 15–19 moderately severe, 20–27 severe. PHQ-9 is known to be superior to previous diagnostic tools for depression in terms of reliability, and time for completion [6]. According to the study of Kurt Kroenke, internal reliability of PHQ-9 was great, with a Cronbach’s α of 0.89. And also, test-retest reliability of the PHQ-9 was excellent [6]. Although cut-off scores are used for PHQ-9 to diagnose depression, higher total scores indicate more severe depression [6].

#### Independent variables

Survey questions for the knowledge of COVID-19 and practice of social and hygienic prevention activities, were selected through expert meetings comprising public health experts at the Yonsei Global Health Center (YGHC), and content validity was confirmed through pre-tests in 10 individuals. The questions were reviewed by local professors and experts in each country to produce questionnaires that are suitable for each country.

##### Knowledge of COVID-19

To assess the level of knowledge of COVID-19 in the survey participants, the following questions were asked. The question “How does COVID-19 spread?” had the following sub-questions: a. droplet (saliva); b. contact through contaminated objects; and c. air. The question “Have you ever heard about these or know these?” had the following sub-questions: (a) number of confirmed COVID-19 cases; (b) number of COVID-19 deaths; and (c) number of recovered COVID-19 cases. Each sub-question could be answered “yes” corresponding to a score of 1 or “no” corresponding to a score of 0. The scores for the six questions were summed to indicate the level of knowledge of COVID-19; the minimum score was 0, the maximum was 6, and the mean was 5.05.

##### Practice of social prevention activities

To assess the participants’ practice of prevention activities in terms of social distancing, the following items were asked: in the past 2 weeks, “I avoided using public transit,” “I refrained from using the elevator,” and “I avoided meetings with 10 or more people.” These items were rated on a 5-point Likert scale as follows: “everyday” corresponding to a score of 5, “mostly” corresponding to 4, “sometimes” corresponding to 3, “rarely” corresponding to 2, and “never” corresponding to 1. These items were used to construct a continuous variable of social prevention activities with a total score of 15.

##### Practice of hygienic prevention activities

To assess the participants’ practice of prevention activities in terms of personal hygiene, the following items were asked: in the past 2 weeks, “I covered my mouth when coughing and sneezing,” “I washed my hands with soap and water,” “I washed my hands immediately after coughing, sneezing, or touching my nose,” “I wore masks often regardless of the presence of symptoms,” and “I washed my hands after touching contaminated objects.” These items were rated on a 5-point Likert scale as follows: “everyday” corresponding to a score of 5, “mostly” corresponding to 4, “sometimes” corresponding to 3, “rarely” corresponding to 2, and “never” corresponding to 1. These items were used to construct a continuous variable of personal hygiene activities with a total score of 25.

##### Stringency index score [7]

To assess each country’s level of response to COVID-19, this study used the stringency index from the Oxford COVID-19 Government Response Tracker developed by Oxford University. Stringency index score calculate by simple averages of the individual items. Stringency index consists of nine items as follows: school closure, workplace closure, public event cancellation, restrictions on gathering size, public transit closure, stay-at-home requirements, restrictions on internal movement, restrictions on international travel, and public information campaigns. The stringency index ranges from 0 to 100, and scores closer to 100 indicate higher levels of response from the government.

##### Social capital

This study used the social capital pillar from the Legatum Prosperity Index, which assesses the level of social acceptance in each country. The pillar consists of five items: personal and family relationships, social networks, interpersonal trust, institutional trust, and civic and social participation. These elements were calculated by 17 indicators weighted differently by the importance of indicators. As the same way, social capital pillar calculated by five elements weighted differently by the importance of elements. The score for social capital ranges from 0 to 100, with scores closer to 100 indicating higher social capital.

### Statistical analysis

This study performed the following statistical analyses to analyze the factors that influence depression in the public during the COVID-19 pandemic. STATA 15.1 used to perform following analysis. First, descriptive analysis was done to see the individual and country characteristics of participants. Second, to confirm the level of depression in the public in each country, the participants’ depression was plotted on a Boxplot graph for analysis. Third, to confirm personal and national factors that influence depression in individuals, a multi-level analysis was conducted. Variance inflation factors (VIF) were calculated to confirm the multicollinearity of multi-level analysis as follows: 3.71 for maximum stringency index score, 1.08 for minimum subjective health, and 2.18 for average. Since VIF was less than 10, there was no multicollinearity. A total of three models were used for the multi-level analysis. Model 0 was used to test for regional differences; simple linear regression was used in Model 1 to assess personal factors that affect depression by excluding regional variables; multilevel mixed-effect model was used in Model 2 to assess complex personal and regional factors and included variables in individual and country level.

### Ethical consideration

This study was approved by the institutional review board of Yonsei University (IRB: 1041849–202,005-SB-057-02).

## Results

Table 3 summarizes the descriptive statistics on personal and regional characteristics of the countries and participants of the survey. The number of participants was highest in Indonesia (19.48%), followed by Peru (13.57%), South Korea (12.66%), the Democratic Republic of Congo (10.34%), the Philippines (10.13%), China (9.88%), Paraguay (6.93%), Ethiopia (6.15%), and Japan (5.20%). There were more female participants (67.16%) than male participants (32.84%). Of the participants, 72.49% reported no experience of discrimination at the national level due to COVID-19. In terms of subjective health, 0.15% thought they were very unhealthy, 0.78% thought they were unhealthy, 16.58% thought they were average, 45.96% thought they were healthy, and 36.27% thought they were very healthy; 82.23% of the participants responded that they were either healthy or very healthy. The mean total PHQ-9 score was 7.07, which was higher than the cut-off of 5 for mild depression.

**Table 3:** Personal and national characteristics of each country and participants

Category/source	N(%)/M ± SD
**Personal characteristics**	
**Number of participants in each country**	
**South Korea**	360(12.66)
**China**	281(9.88)
**Japan**	148(5.20)
**Philippines**	288(10.13)
**Indonesia**	554(19.48)
**Peru**	386(13.57)
**Paraguay**	197(6.93)
**Ethiopia**	175(6.15)
**Democratic Republic of Congo**	294(10.34)
**Sex**	
**Male**	881(32.84)
**Female**	1802(67.16)
**Marital status**	
**Not married**	2346(87.44)
**Married**	337(12.56)
**Number of family members**	
**One-person household**	248(9.24)
**2**	334(12.45)
**3–5**	1574(58.67)
**6 or more**	527(19.64)
**Experience of discrimination at the national level due to COVID-19**	
**No**	1945(72.49)
**Yes**	738(27.51)
**Subjective health**	
**Very unhealthy**	4(0.15)
**Unhealthy**	21(0.78)
**Average**	452(16.58)
**Healthy**	1233(45.96)
**Very healthy**	973(36.27)
**Trust in hospitals**	
**Very distrustful**	115(4.29)
**Distrustful**	504(18.78)
**Trustful**	1288(48.01)
**Very trustful**	526(19.6)
**Unknown**	250(9.32)
**Private health insurance**	
**No**	1238(43.53)
**Yes**	1445(56.47)
**Age**	
**Age (M±SD)**	25.71 ± 8.55
**Knowledge of COVID-19**	
**Total score for knowledge (M±SD)**	5.05 ± 1.10
**Practice of social prevention activities**	
**Social prevention activities Total score (M±SD)**	9.84 ± 4.29
**Practice of hygienic prevention activities**	
**Hygienic prevention Activities total score (M±SD)**	18.28 ± 6.66
**Level of Depression**	
**Sum of PHQ-9 Score(M±SD)**	7.07 ± 6.12
**National characteristics**	
**Stringency index score**	
**Oxford Stringency Index**	77.79 ± 12.30
**South Korea**	77
**China**	57
**Japan**	56
**Philippines**	93
**Indonesia**	71
**Peru**	92
**Paraguay**	92
**Ethiopia**	75
**Democratic Republic of Congo**	81
**Life expectancy**	
**World Bank**	73.35 ± 6.69
**South Korea**	82.63
**China**	76.47
**Japan**	84.10
**Philippines**	70.95
**Indonesia**	71.28
**Peru**	76.29
**Paraguay**	73.99
**Ethiopia**	65.87
**Democratic Republic of Congo**	60.03
**Literacy rate**	
**Our World in Data**	91.02 ± 12.63
**South Korea**	97.96
**China**	96.35
**Japan**	99
**Philippines**	96.61
**Indonesia**	95.43
**Peru**	94.37
**Paraguay**	95.53
**Ethiopia**	49.03
**Democratic Republic of Congo**	77.22
**Social capital**	
**Legatum Prosperity Index**	53.01 ± 12.34
**South Korea**	42.482
**China**	57.248
**Japan**	44.448
**Philippines**	59.318
**Indonesia**	73.431
**Peru**	42.345
**Paraguay**	53.697
**Ethiopia**	46.814
**Democratic Republic of Congo**	38.740
**PPP (1 000 000 USD)**	
**World bank**	3 850 000 ± 6 780 000
**South Korea**	2 029 000
**China**	23 160 000
**Japan**	5 429 000
**Philippines**	875 600
**Indonesia**	3 243 000
**Peru**	424 400
**Paraguay**	68 330
**Ethiopia**	200 200
**Democratic Republic of Congo**	28 880

*PPP* Purchasing Power Parity, *N* Number of cases, *M* Mean, *SD* Standard Deviation

**Fig. 1:**
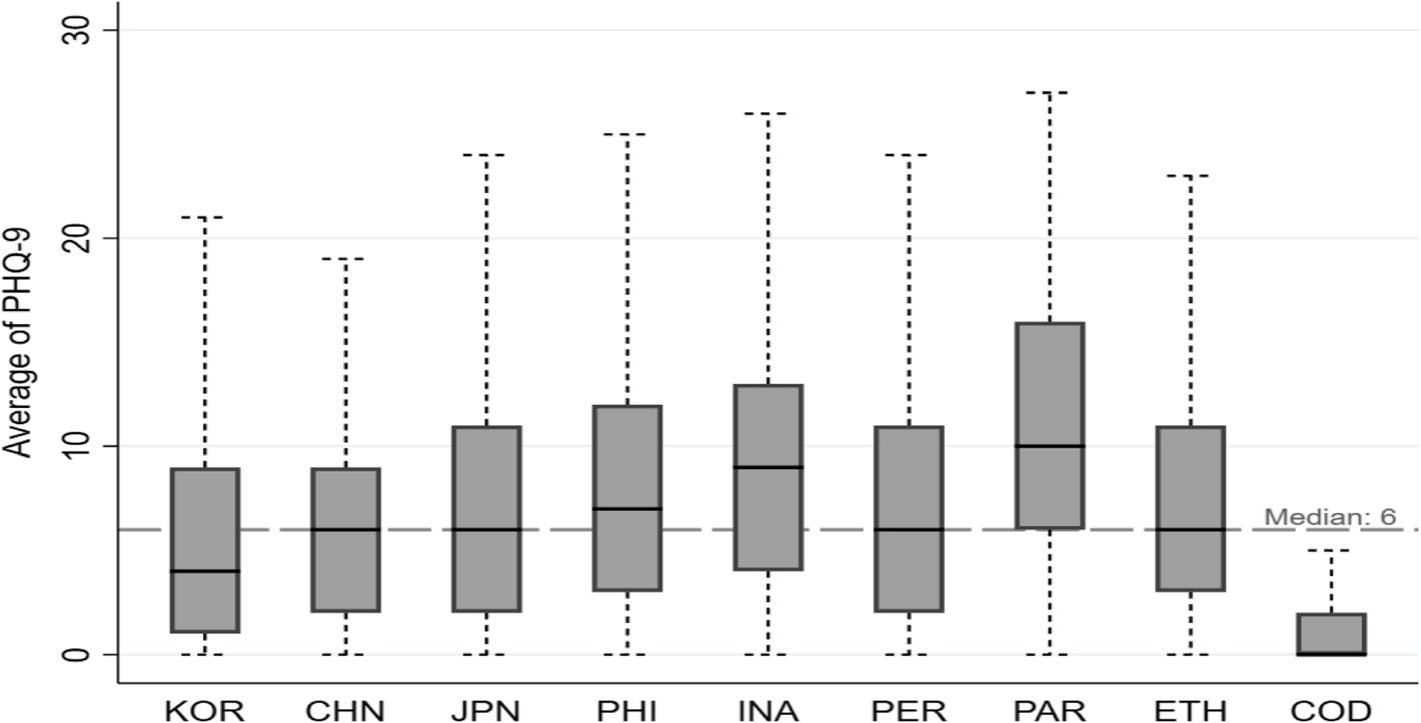
Distribution of depression by countries

Figure 1 is a boxplot graph showing the distribution of depression in each country. The median score for all participants was 6. The median was higher than the overall median in the Philippines, Indonesia, and Paraguay, suggesting a higher level of depression. The median score was 4 and 0 in South Korea and the Democratic Republic of Congo, respectively; these were lower than the overall mean, suggesting a lower level of depression.

Table 4 shows the results of multi-level regression analysis. Model 0 had no independent variable, and intraclass correlation coefficients (ICC) was 0.130 for the model. This indicated that approximately 13.0% of variance in participants’ depression was due to regional differences. Since ICC over 5% is interpreted as significant regional differences in social sciences, the use of multi-level analysis was acceptable in this study.

**Table 4:** Results of multi-level analysis

Category	Model 0		Model 1		Model 2	
			Coef.	95%CI	Coef.	95%CI
**Personal variable**						
**Sex**						
Male			(ref)		(ref)	
Female			0.720**	(0.253, 1.186)	0.718**	(0.248, 1.181)
**Age**			−0.090***	(−0.122, − 0.058)	−0.086***	(− 0.118–0.054)
**Marital status**						
Not married			(ref)		(ref)	
Married			−0.256	(− 0.054, 1.840)	− 0.222	(− 0.949, 0.504)
**Number of family members**						
One-person household			(ref)		(ref)	
2			0.893	(− 0.054, 1.840)	0.847	(−1.001, 1.794)
3–5			−0.149	(− 0.930, 0.632)	− 0.249	(−1.028, 0.530)
6 or more			0.083	(−0.799, 0.966)	0.034	(−0.849, 0.917)
**Experience of discrimination at the national level due to COVID-19**						
No			(ref)		(ref)	
Yes			0.989***	(0.498, 1.479)	0.948**	(0.458, 1.436)
**Subjective health**			−1.624***	(− 1.921, − 1.325)	−1.612***	(− 1.909, − 1.315)
**Knowledge of COVID-19**			0.062	(−0.141, 0.265)	0.074	(− 0.129, 0.277)
**Trust in hospitals**			−0.216**	(−0.374, − 0.057)	−0.231**	(− 0.388, − 0.072)
**Private health insurance**						
No			(ref)		(ref)	
Yes			−0.233	(−0.783, 0.316)	−0.370	(− 0.915, 0.176)
**Practice of social prevention activities**			−0.004	(−0.081, 0.072)	− 0.002	(− 0.078, 0.073)
**Practice of hygienic prevention activities**			−0.208***	(−0.271, − 0.143)	−0.228***	(− 0.289, − 0.166)
**National variables**						
**Stringency index score**					0.139***	(0.074, 0.203)
**Life expectancy**					0.453***	(0.333, 0.573)
**Literacy rate**					−0.096***	(−0.152, − 0.040)
**Social capital**					0.230***	(0.173, 0.286)
**PPP**					5.86	(−5.60, 1.73)
**ICC**	0.130		0.221		0.016	

*p < 0.05,**p < 0.01;*** < *p* < 0.001

*PPP* Purchasing Power Parity, *ICC* Interclass Correlation Coefficient, *Coef*: Regression Coefficients, *95%CI* 95% Confidence Interval

Adjusted R-squared score of the Model 1(linear regression model) was 0.0441. Personal variables were added to model 0 to construct model 1, which demonstrated that depression was higher in females than males and in participants who experienced discrimination due to COVID-19 than in those who have not. In contrast, depression was lower in older participants, those with good subjective health, and those who practiced personal hygiene for prevention.

Likelihood ratio test value of model 2(Multilevel mixed-effects model) was 16.65 and the *P*-value was 0.000. National variables were added to model 1 to construct model 2. In this model, the results were identical to those of model 1 in terms of personal variables. The model also revealed that depression was higher when the stringency index score was higher, when life expectancy was higher, and when social capital was higher. In contrast, depression was lower when the literacy rate was higher.

## Discussion

To identify the factors that affect depression in the public during the COVID-19 pandemic, this study performed analyses at national and personal levels.

The first goal of the study was to assess the level of depression in the public in the nine surveyed countries. The level of depression in the public was particularly high in the Philippines, Paraguay, and Indonesia. According to the Institute for Health Metrics and Evaluation in 2017, the mean percentage of the population with depressive disorders compared to the population between 25 and 29 years of age in the nine countries was 3.47%, which was lower than the mean in the Philippines (2.50%), Indonesia (2.46%), and Paraguay (3.40%). These findings contrast with our results of depression in each country. Such differences may be attributed to the COVID-19 pandemic because social distancing and stay-at-home orders implemented by governments to prevent the spread of COVID-19 are associated with increased social isolation. This study confirmed previous findings that such social isolation influences mental health in young and middle-aged individuals, such as loneliness, depression, and stress [8]. The COVID-19 pandemic has also significantly influenced world economies; on April 29, the International Labor Organization reported that one out of six young workers lost their jobs due to COVID-19 [9]. Regarding such unemployment, Olesen claimed that loss of job influences mental health, whilst poor mental health can also lead to unemployment [10]. Although the participants included in this study did not cover all age groups and various factors influence depression in individuals, changes observed in the last 3 years would have been significantly influenced by the COVID-19 pandemic.

The second aim of this study was to confirm the personal factors that influence depression in individuals. In this study, the following personal variables were found to influence depression in individuals: sex, age, experience of discrimination at the national level due to COVID-19, trust in hospitals, and practice of personal hygiene for prevention. Our findings regarding sex are in line with previous reports that depression is higher in females than males [11]. Depression was higher when the participants were younger. Considering the mean age and standard deviation of participants, this finding aligns with previous findings that depression is higher in younger individuals in their 20s and 30s [12].

This study also confirmed that depression was higher in the group who experienced discrimination at the national level due to COVID-19 than in the group who did not. Shortly after the discovery of COVID-19, the disease was initially named “Wuhan pneumonia” or “Wuhan virus,” which led to worldwide discrimination against China. Matteo Salvini, the former deputy prime minister of Italy associated African asylum seekers with COVID-19 and demanded the closure of the country’s borders [13]. On February 24, Jonathan Mok, a Singaporean international student in the UK, faced physical violence from attackers who stated, “We don’t want your coronavirus in our country” [14]. On March 25, a Bangladeshi male with suspected symptoms of COVID-19 committed suicide from fear of discrimination from other towns and a confirmed diagnosis [15]. As such, discrimination is widespread worldwide during the COVID-19 pandemic, and many people have been reporting psychological pain due to this, including depression and anxiety disorder. Therefore, on February 11, WHO recommended refraining from the use of discriminatory terms, such as Wuhan virus or Wuhan pneumonia, rather using neutral terms such as COVID-19 or coronavirus as the official name. To overcome the COVID-19 pandemic, everyone around the world should have a sense of common belonging and contribute to preventing a 2nd pandemic wave through cooperation rather than discrimination. Moreover, shared efforts must be made to develop vaccines, treatments, and new drugs.

We found depression was lower in the group that actively practiced personal hygiene for prevention. Accordingly, countries will need to implement continued COVID-19 public information campaigns to publicize the accurate route of infection of COVID-19 and correct prevention efforts. Further, public health education will need to recommend prevention activities to prevent further spread of COVID-19 and decrease depression in the public.

The third aim of the study was to confirm national factors that influence depression in individuals. The following national variables were found to have significant effects on depression in individuals: stringency index score, life expectancy, literacy rate, and social capital. Regarding stringency index scores, depression was higher in countries with higher scores. Although national responses are necessary in preventing the spread of COVID-19, unnecessarily stringent responses may cause depression, anxiety, and tension in the public. To prevent these issues, national psychological prevention strategies are necessary. Accordingly, countries should establish online and remote mental health support programs to alleviate anxiety in the public through contactless means. Online and remote mental health support programs are non-face-to-face mental health counseling institutions that utilize the Internet and phone connection. In South Korea, an integrated mental health support program for COVID-19 was established under the national trauma center, and the program provides free mental health counseling. Non-government groups, such as the Korea Psychological Association, also offer similar services. Through these services, approximately 334,000 individuals received remote psychological counseling as of June 3. Active introduction of social prescribing is also necessary at the national level. Social prescribing refers to the prescription of various non-clinical activities, such as volunteer activities, art activities, group learning, gardening, making friends, cooking, and various sports activities. In the UK, the NHS introduced social prescribing in 2016 [16]. Matt Hancock, the Secretary of State for Health and Social Care in the UK, stated that long-term solutions are needed for the COVID-19 crisis and a solution is the national efforts for social prescribing, at the National Academy for Social Prescribing (NASP) on March 12 [17]. On the same day, Helen Stokes-Lampard, the Chair of NASP, also emphasized the need for social prescribing stating that the sense of community should be strengthened these days when social distancing is in place [18]. In South Korea, some claim that the role of health teachers should be expanded to include infectious disease in addition to the current scope of non-infectious diseases and that positions such as social prescribers should be included to broaden the scope of work [19].

This study also found that depression decreased with increasing literacy rate, which coincides with the findings of previous studies [20, 21]. Nutbeam argued that low literacy leads to poor health, and Gazmararian confirmed that the prevalence of depressive symptoms was 2-fold higher in the group with low health literacy than in the group with higher health literacy. Accordingly, in the short-term, countries will need to strive to provide health information and education on COVID-19 to promote and recover the mental health of those affected by COVID-19. And also, government should aware the spread of pseudoscientific information. In the study of Escolà-Gascón, irrational beliefs in pseudoscientific information grew after the first months of social quarantine [22]. This kind of pseudoscientific information could cause infodemic, and accurate information campaign of COVID-19 could be the main keypoint to tackle infodemic. Moreover, in the long-term, quality education, which is the 4th Sustainable Development Goals, should be provided to satisfy the public demand for education, increase health literacy, promote mental health in the public through this, and contribute to sustainable development Goals.

This study also found that depression was higher with higher social capital. This result contrasts with previous findings that social capital or social bonding had positive effects on mental health [23, 24]. The social capital pillar of the Legatum Prosperity Index used in this study consists of personal and family relationships, social networks, interpersonal trust, institutional trust, and civic and social participation. Citizens of countries that scored high in these items would tend to enjoy social engagements. However, due to the COVID-19 pandemic, physical and social distancing has been in place for a long time. As a result, the participants would have experienced social disconnection, decreasing their satisfaction from social activities, and increasing depression. To address this problem, government should take effort to make healthy untact culture. Such as on-line concert, video game, E-museum, video conference or video call and so on. This new form of culture could make people feel like new type of connection than social disconnection.

### Limitations

There are a few limitations to this study. First, due to the COVID-19 pandemic, this study was conducted through contactless online surveys. According to Heiervang, who examined the advantages and limitations of web-based surveys, web-based surveys are more affordable and faster but also incomplete due to the absence of an interviewer [25]. Moreover, since web-based surveys can only be used in populations who can access the internet and read, there could be bias in sampling. We could handle this bias by surveying through telephone and mail, or by face-to-face survey when the pandemic ends. Second, this cross-sectional study is limited in assessing the effects of the COVID-19 pandemic over time. Therefore, future studies will need to verify the effects of the COVID-19 pandemic on depression in each country over time. Third, since the total PHQ-9 score was the dependent variable in the study, the results cannot be generalized to all countries. For instance, according to one study by Urtasun investigating the validity of PHQ-9 in Argentina, the cut-off PHQ-9 scores should be adjusted as follows in Argentina: 6 for mild depression, 9 for moderate depression, and 15 for severe depression [26]. As such, due to differences across countries, the generalization of our findings may be limited in some countries.

## Conclusion

This study aimed to identify the effects of personal and national factors on depression in individuals during the COVID-19 pandemic.

First, the median score for depression was higher than the overall median of 6 in the Philippines, Indonesia, and Paraguay whereas South Korea and the Democratic Republic of Congo had scores below the overall median.

Second, discrimination at the national level due to COVID-19 and personal hygiene for prevention were notable personal factors influencing depression in individuals. Depression was higher in the group who experienced discrimination at the national level due to COVID-19 than in the group who did not, and depression was lower in the group who practiced personal hygiene for prevention than in the group who did not.

Third, at the national level, social capital and stringency index score were major factors influencing depression in individuals. In contrast to previous reports, depression was higher in participants living in countries with high social capital than in those living in countries with low social capital. This result indicates that individuals with high social capital who enjoy social activities experienced notable increases in depression due to social distancing during the COVID-19 pandemic.

The findings suggest that each country should establish detailed guidelines for each quarantine level considering the national and personal factors to implement appropriate quarantine policies. Depression was higher in participants living in countries with higher stringency index scores than in those living in other countries. Maintaining a high level of response for safety cannot be criticized. Regarding social distancing, a previous study reported a high correlation between traffic load and the number of confirmed COVID-19 cases [27]. However, in the current situation in which coexisting with COVID-19 has become inevitable, inflexible and stringent policies not only increase depression in the public but may also decrease resilience to COVID-19 and delay preparations for coexistence with COVID-19. Accordingly, when establishing policies such as social distancing and quarantine, each country should implement the psychological characteristics of the public, such as practices of prevention activities and experiences of discrimination to minimize the negative effects of COVID-19 policies.

## Data Availability

The datasets used and/or analyzed during the current study are available from the corresponding author on reasonable request.

## Abbreviations

WHO: World Health Organization
NHS: National Health Service
US: United States
EU: European Union
PHQ-9: Patient Health Questionnaire-9
VIF: Variance inflation factors
ICC: Intraclass correlation coefficients
UK: United Kingdom
NASP: National Academy for Social Prescribing
